# Anxiety

**Published:** 1890-04

**Authors:** 


### ANXIETY.

Anxiety about present troubles or about prospective difficulties never brought any good to those who indulged in it. Those who have succeeded in life and enjoyed it most have been the people who were buoyant in spirit, and who resolutely refused to allow the cares of life to unduly depress them. Of course some persons have a constitutional tendency to despondency, and they can sometimes see a cloud when there is none ; but with most it is simply a matter of exercising the will. Instead of allowing the mind to brood over things that cannot be helped, it should be set to work upon the duty that lies nearest to it If we would only make up our minds to look at the bright side of things oftener, the cares that are now almost crushing the hope out of us would lose half their power, Worrying about matters does not improve them in the least ; on the contrary, it weakens the purpose, robs the physical nature of its vitality, and totally unfits us to cope with the obstacles that lie in our path. As for meeting trouble half way, this is one of the most foolish of practices. It often happens that the troubles to which we look forward with such heavy foreboding either do not come at all, or are not so terrible when we meet them as we feared they would be. The best corrective for an anxious, fretful spirit is to do one's duty faithfully in his own station in life, and to trust in Providence for strength and guidance in times of trouble and peril.

